# Sequelae lesions in the larynxes of patients with Paracoccidioidomycosis

**DOI:** 10.1590/S1808-86942011000100007

**Published:** 2015-10-19

**Authors:** Jose Mauricio Lopes Neto, Leonardo Muller Severo, Rinaldo Poncio Mendes, Silke Anna Thereza Weber

**Affiliations:** 1 3rd year resident in otolaryngology - University Hospital of the Medical School of Botucatu; 2 3rd year resident in otolaryngology - University Hospital of the Medical School of Botucatu; 3 PhD, Full Professor - Department of Infectous Diseases of the Medical School of Botucatu; 4 PhD, Assistant Professor - Department of Ophthalmology, Otolaryngology and Head and Neck Surgery - Medical School of Botucatu-UNESP

**Keywords:** dysphonia, paracoccidioidomycosis, mycosis, laryngeal diseases, larynx

## Abstract

Paracoccidioidomycosis (PCM) is a systemic disease that in its aftermath form is characterized by clinical manifestations related to anatomic or functional sequelae of organs and systems affected during the period of state.

**Aim:** To describe the anatomical and functional sequelae in patients with treated laryngeal PCM.

**Materials and Methods:** Retrospective study. We reviewed the charts from of 49 male patients, aged between 30 to 60 years, diagnosed with laryngeal PCM during the period of 1999 to 2004. In all patients the diagnosis of PCM was confirmed by demonstration of the fungus in sputum, cytological or histopathological examination and being followed up by the Infectious and Parasitic Diseases Department.

**Results:** The vocal folds were the most affected laryngeal structure, being affected in 67% of the patients. The epiglottis and the aryepiglottic folds were affected in 55% and 53% of the cases, respectively. Vestibular folds were changed in 46% of the patients. In 40% of the cases there were changes in the arytenoids. During phonation, 28% of the patients showed limited movement of the vocal folds, unilateral vocal fold paralysis was found in 4%. 24% of the cases had glottic lumen reduction and 4% showed glottic stenosis, 2% needed tracheostomy.

**Conclusion:** Sequela lesions of the laryngeal PCM are extensive and cause functional limitations in most cases.

## INTRODUCTION

Paracoccidioidomycosis (PCM) is a systemic mycosis caused by the *paracoccidioides brasiliensis* thermodymorhic fungus, which was first described in 1908 by Adolfo Lutz^1^; later on, in 1912, Afonso Splendore suggested the name *Zymonema brasiliense*; and in 1930, Floriano de Almeida proposed the current name. It is geographically distributed in the Americas only, from Mexico down to Argentina, and it is the most frequent systemic mycosis present in Latin America. It is believed that there are around 10 million people infected with it, and that 2% will develop the disease. It is estimated an annual incidence of three new cases for every 100,000 inhabitants^2,^^3^.

The lungs are affected through inhaling the spores, and this is the main way of acquiring the disease. There is then an alveolar reaction (pneumonitis) and fungal spread through the lymphatic tissue to the paratracheal and parabronchi lymph nodes, forming the ganglion-pulmonary primary complex^2,^^4^. There is also direct spore inoculation through breaks in the skin and mucosas.

The chronic spread form of PCM is the most common, and in general it affects rural workers after their fourth decades of life, at a men/women ratio of 15:1. The patients have a long and slow history of symptoms, upper airway mucosa, skin and adrenal glands. There may be adenopathy, but in general this is not the predominant finding^2^.

In studies assessing patients with acute PCM, in up to 42% of the cases we have laryngeal involvement, the vocal folds and epiglottis are the most affected structures^5^, ^6^, ^7^, ^8^.

Machado Filho et al. (1960)^5^ carried out a study in patients with confirmed PCM, including routine indirect laryngoscopy. They assessed 104 cases, and in 40.6% there were laryngeal lesions, of which 15.0% did not have symptoms.

Fernandes et al. (1986)^6^ followed 56 cases of PCM and found laryngeal involvement in 42.8%. They stressed that these patients not always complained of hoarseness.

Bastos et al. (2001)^7^ assessed 17 PCM cases with laryngeal involvement as to their complaints and structures involved. Before treatment onset, 58.8% of the patients had dysphonia. The most frequently involved structures were the vocal folds (64.7%) and the epiglottis (47%). There are no reports concerning the persistence of dysphonia.

As to the PCM sequela, the symptoms are associated with anatomical or functional changes in the organs and systems affected during the disease period and that, after treatment, show fibrotic scars. Thus, it is common to have dyspnea because of fibrosis and pulmonary emphysema, hoarseness, trachea and laryngeal stenosis and Addison's disease^9^, ^10^, ^11^, ^12^, ^13^, ^14^.

There are very few studies involving patients with sequela lesions because of laryngeal PCM. One of the pioneering studies was developed by Machado et al. (1965)^11^ which assessed the sequelae of 579 patients with PCM, followed by a period of six months to nine years. They stressed the importance of glottal or tracheal stenosis, seen in 23.9% of the patients with laryngeal involvement.

More recently, Valle et al. (1995)^12^ carried out an endoscopic study in 80 patients with sequela PCM. Of the 30 patients with laryngeal involvement, six required tracheotomy.

In 2006, Weber et al. (2006)^8^ assessed 50 individuals, 35 with a diagnosis of PCM. In that study, they formed groups made up of patients with pulmonary PCM in its sequela phase and laryngeal PCM in the sequela and active phases, and they were compared with the control group as to the degree of dysphonia and laryngeal anatomic changes during the endoscopic exams.

The number of patients with sequela caused by the PCM is still high, and the complaint of dysphonia is common. The ENT physician must be familiarized with the aspect of the lesions in order to be able to diagnose it early on and to educate the patient and the infectologist.

## OBJECTIVES

To describe the laryngeal functional and anatomical changes in patients with paracoccidioidomycosis in its sequela phase.

## MATERIALS AND METHODS

The study was approved by the Ethics in Research Committee of our institution, under protocol # 375/99. All the patients signed a Free and Informed Consent Form (FICF).

This is a retrospective, cross-sectional study, done by analyzing the medical charts and the images of patients with sequela PCM recorded in a video home system (VHS). The endoscopies were carried out between 1999 and 2004, and this was the time interval proposed to start the project in 1999. At the time of the endoscopy, all the patients had already been submitted to medical treatment for PCM for at least six months and carried out an endoscopic exam to control lesion healing. The PCM diagnosis was done during the active disease stage, confirmed by showing the fungus in the sputum, cytological or histopathological exam.

We took off the study patients with other concurrent infectious diseases such as HIV, tuberculosis, leishmaniasis, amongst others - data taken from their medical charts. We also took off those patients diagnosed with laryngeal neoplasia or those who underwent previous laryngeal surgery or radiotherapy.

From the medical charts we obtained the demographic data (age, gender) and the complaints of hoarseness and dysphagia (main complaints from functional sequela changes).

The endoscopies were carried out in the Endoscopy Department of the University Hospital of the Medical School of Botucatu. The exam was carried out with the patient seating down, awoke, and submitted to nasal topical anesthesia with 4% lidocaine. We used the 4.2mm Storz flexible nasal fiberscope. The images were captured by a Storz camera and recorded after proper authorization in written by the patients.

We analyzed the endoscopic images stored in VHSe; the larynx was assessed as to its anatomical components (epiglottis, vestibular fold, vocal fold, arytenoid and posterior commissure) and functional (as to the movement during phonation and breathing).

As to the anatomical changes found, they were described as follows:

1. Edema: increase in the mucosa mass, painless and without color change.
2. Hyperemia: change of the normal mucosa color to a reddish color.
3. Fibrous thickening: any denser area of the mucosa which would suggest increase in consistency.
4. Amputation.
5. Change in shape.
  We also noticed the phonation behavior (vocal fold coaptation) and salivary stasis in the pyriform sinuses.

## RESULTS

During the study period, we included 49 male patients, in the age range between 30 and 60 years, with mean age of 43.4 years, diagnosed with PCM under outpatient follow up by the Department of Parasitic and Infectious Diseases.

All the patients complained of hoarseness or there was dysphonia/hoarseness described in the patient's chart. Many patients had reports of dysphagia; nonetheless, there was no specific assessment on possible difficulties in swallowing foodstuff (solids and liquids), cough after swallowing, and need for maneuvers during feeding (as head anti-flexion).

There were lesions in multiple laryngeal segments in 35 (71%) of the 49 patients assessed. The most frequently affected structures were the vocal folds, followed by the epiglottis and the arytenoids (Table 1).

**Table 1:** Compromised laryngeal structures according to frequency.

Laryngeal structure	Number of patients affected
Vocal folds	33 (67%)
Epiglottis	27 (55%)
Aryepiglottic folds	26 (53%)
Vestibular folds	23 (46%)
Arytenoids	20 (40%)

**Figure 1:**
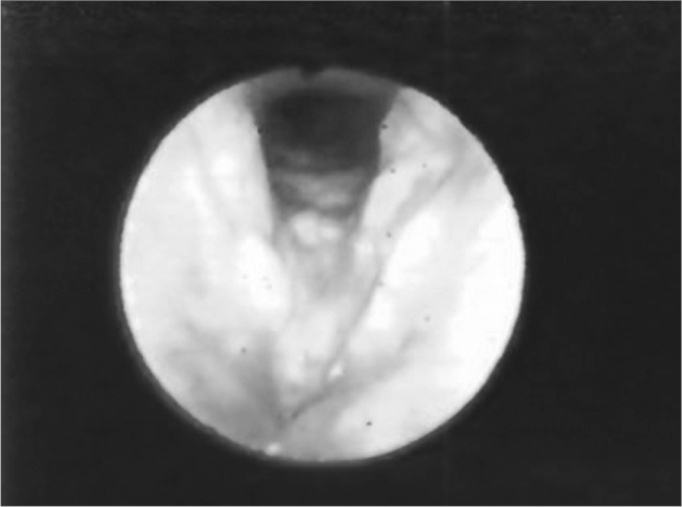
Hyperemia with vocal fold thickening.

**Figure 3:**
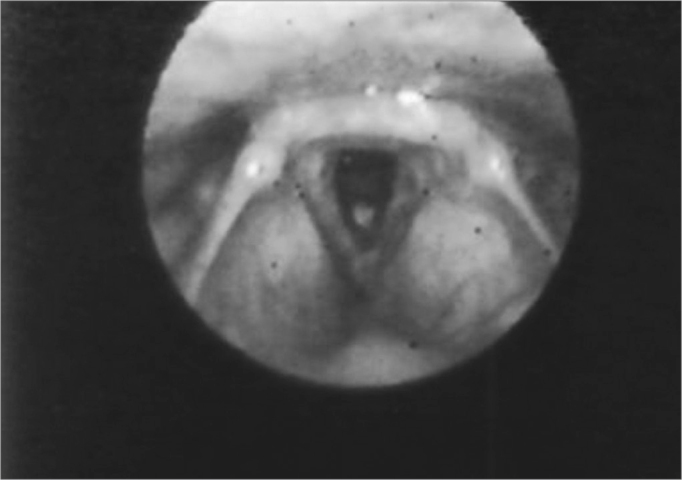
Vocal fold thickening with free border irregularities and interarytenoid edema.

**Figure 4:**
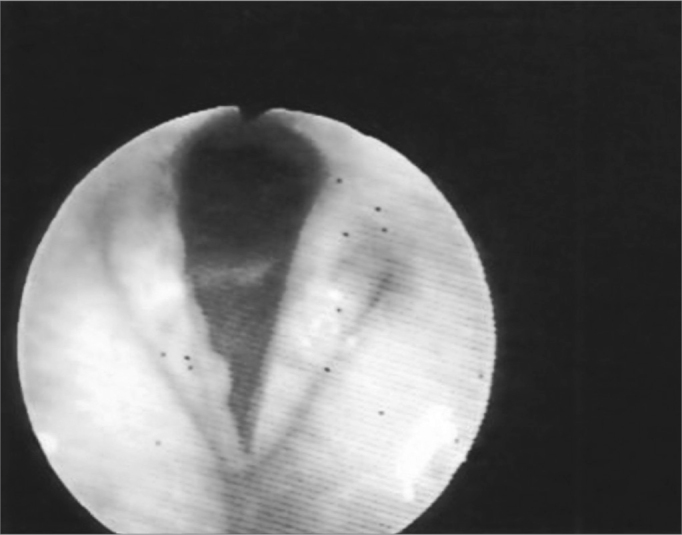
Thickening with vocal fold hyperemia and free border irregularities.

The vocal folds were the most affected laryngeal structures - in 33 (67%) patients there were lesions. Thus, we had a fibrous thickening in 27 (55%) of them, hyperemia in 14 (28%), irregular border contour in 13 (26%) and edema in 8 (16%). (Figs. 1, 3 and 4). (Tables 1 and 2).

**Table 2:** Distribution of sequelae lesions in laryngeal structures.

	Hyperemia	Edema	Thickening	Amputation	Change in shape
Epiglottis	-	27 (55%)	-	23 (46%)	16 (32%)
Vocal folds	14 (28%)	8 (16%)	27 (55%)	-	13(26%)
Aryepiglottic folds	-	16 (32%)	-	-	17 (34%)
Vestibular folds	17 (34%)	12 (24%)	-	-	5 (10%)
Arytenoids	4 (8%)	20 (40%)	-	-	-

**Figure 2:**
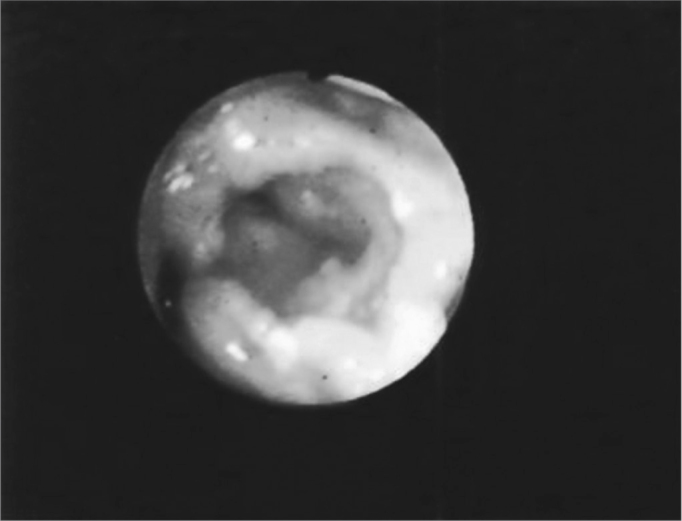
Edema with interarytenoid region hyperemia. Epiglottis hyperemia with partial amputation.

**Figure 5:**
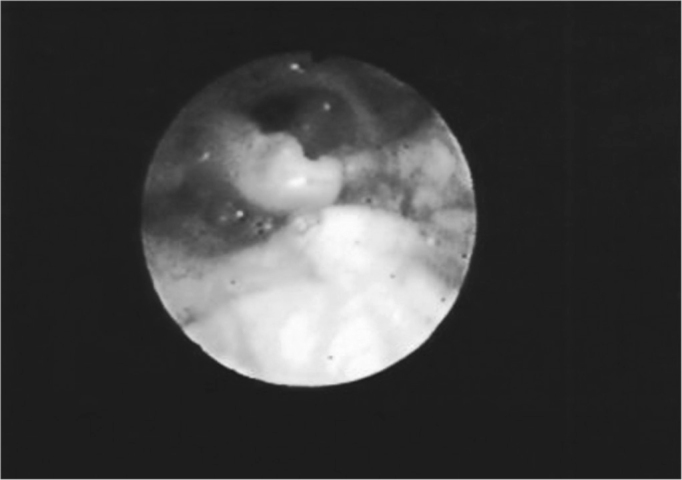
Partial epiglottis amputation.

The second most affected structure was the epiglottis - 27 (55%) patients. There were shape changes in 16 (32%) patients, amputation in 23 (46%) patients and edema in 27 (55%). (Figs. 2 and 5) (Table 2).

The laryngeal structures affected were described in their frequency (%) of the most commonly found lesion per structure.

In the aryepiglottic folds, there were changes in 26 (53%) of the cases assessed. The change in shape was present in 17 (34%) patients and edema in 16 (32%) (Table 2).

As to the vestibular folds, we noticed changes in 23 (46%) patients. We found edema in 12 (24%), hyperemia in 17 (34%) and shape changes as atrophy in 5 (10%) patients (Table 2).

In 20 (40%) cases we found changes in the arytenoids, and edema was present in 20 (40%) and hyperemia in 4 (8%) patients. (Figs. 2 and 3).

During phonation, there was movement limitation in the vocal folds of 14 (28%) patients and unilateral paresis in 2 (4%) cases. Lumen reduction in the supraglottal region was seen in 12 (24%) patients and glottal stenosis in two (4%) patients, and one (2%) required tracheostomy.

We noticed salivary stasis in at least one pyriform sinus in 10 (20%) patients, and all of them had laryngeal movement restrictions upon exam. Nonetheless, there had been no prior specific assessment of dysphagia in these patients.

## DISCUSSION

PCM still is an endemic disease in many regions of Brazil, affecting mainly rural workers. In the present paper, all the patients were young males, when they are economically important. Although there are many women performing activities in rural areas (as sugar cane cutting, for example) the frequency of chronic cases is still low among females and it is associated with the inhibitory action of the estrogen on the transformation of the fungus as mycelium (infecting form) to that of yeast (parasitic phase)^15^, and such fact would explain the lack of female patients in our study.

The first report of a laryngeal involvement by the *P. brasilienses* is found in the pioneering work from Lutz (1908)^1^ who, besides reporting the presence of the lesion in the oral mucosa, reported on the second case, the clinical manifestation of hoarseness and the intense laryngeal involvement caused by the paracoccidioides under autopsy.

The endoscopic study revealed involvement of more than one laryngeal structure in 72% of the patients. Other authors have already described the intense involvement, and Machado et al (1965)^11^ reported the largest group of patients involved, they assessed the PCM sequelae in 579 patients, stressing that 16 of these had glottal or tracheal stenosis. By the same token, do Valle et al. (1995)^12^ stress that from 30 patients with laryngeal lesion, six required tracheostomy. Although dysphonia has been reported, only stenosing lesions were described. In our study, 24% of the patients had narrowing of their supraglottal lumen, and 4% had glottal stenosis, and one patient (2%) required tracheostomy. The extension to multiple laryngeal segments and the high incidence of supraglottal lumen narrowing, show that the laryngeal involvement was extensive upon the diagnosis, as per reported by other authors^5^, ^6^, ^7^, [Bibr bib8][Bibr bib13][Bibr bib14].

The dysphonia reported by many authors^1,^^5,^^6,^^11,^^12,^^14^ is not a symptom routinely investigated in all the patients. Fernandes & Fernandes (1986)^6^ emphasized that hoarseness does not have to be present and the laryngeal involvement could be higher than the 42% found in their study. Weber et al. (2006)^8^ compared the degree of dysphonia in patients with laryngeal and/or pulmonary sequela caused by PCM with a control group. They showed that high levels of dysphonia are found in patients with PCM and most of the times with important social impairments. In our study, most of the patients complained of hoarseness, although it was not possible to analyze the social repercussion caused by this symptom.

Of the patient with laryngeal mobility restriction (28%), many had salivary stasis in their pyriform sinuses (71%). This can be one of the factors involved in repetitious pulmonary infections in patients with PCM^2,^^5,^^6,^^10,^^11^. Nonetheless, in this study we did not include specific anamneses of dysphagia in ENT consultations.

Few papers^7^, ^8^, [Bibr bib9][Bibr bib11][Bibr bib12] describe the laryngeal sequelae caused by *P. brasilienses*, showing that there is a greater concern in studying paracoccidioidomycosis in its time of activity. The assessment of the sequelae is very important because of the increasingly longer survival of these patients with treatment available^2,^^7^ and the reinsertion of these patients in their social environment and in the economic activity.

## CONCLUSION

The laryngeal sequelae lesions caused by *P. brasilienses* are extensive and cause functional restrictions in most of the cases. We noticed that the vocal folds are the most affected structures with thickening, and it is the most common sequela change. Moreover, all patients had dysphonia. Other functional changes, such as dysphagia must also be investigated. The otolaryngologist must be familiarized with the sequelae lesions and their functional repercussions in PCM patients.
